# The “pharmakon” of prevention: artificial immunity in the covid-19 pandemic

**DOI:** 10.1590/S0104-59702024000100055

**Published:** 2024-10-14

**Authors:** Sofia Varino

**Affiliations:** i Postdoctoral research fellow, University of Potsdam. Potsdam – Berlin-Brandenburg – Germany; varino.sofia@gmail.com

**Keywords:** Immunity, Prevention, Pandemic, Anthropocene, Covid-19, Imunidade, Prevenção, Pandemia, Antropoceno, Covid-19

## Abstract

The concept of *pharmakon* encapsulates the paradoxical ambivalence of any therapeutic intervention’s harmful as well as beneficial effects; within the context of immunological practices related to covid-19, this ambivalence has been successfully exploited by anti-vaccination movements and is also evident in widespread vaccine hesitancy in wealthy countries where vaccines for this virus are widely available. Here we engage with the theoretical apparatus of the *pharmakon* to examine how care, harm, risk, and prevention are enacted in covid-19 prevention measures and mobilize the transdisciplinary methodologies of science and technology studies to investigate how anticipatory imaginaries drive cutting-edge research on covid-19 vaccines and the clinical and social practices they have elicited.

## Resumo

O conceito de pharmakon abrange a ambivalência paradoxal dos efeitos nocivos e benéficos de qualquer intervenção terapêutica. No contexto das práticas imunológicas relacionadas à covid-19, essa ambivalência tem sido explorada com sucesso pelo movimento antivacina e também é evidente na hesitação vacinal generalizada em países ricos, onde vacinas contra essa doença estão amplamente disponíveis. Aqui, examinamos o aparato teórico do pharmakon para verificar como o cuidado, o dano, o risco e a prevenção são implementados nas medidas de prevenção da covid-19 e mobilizamos metodologias transdisciplinares dos estudos de ciência e tecnologia para investigar como imaginários antecipatórios impulsionam a pesquisa de ponta sobre as vacinas contra a covid-19 e as práticas clínicas e sociais que elas suscitaram.

During the relatively short history of covid-19, biomedical practices of prevention have played a major role in containing the effects of the pandemic. Without a targeted course of treatment, preventing the spread of the virus became a priority for governments and public health agencies including the World Health Organization (WHO) from the start. After March 2020, isolation, quarantine, (social) distancing, and eventually lockdown measures were adopted to various degrees in hopes of minimizing impacts by controlling the propagation of the virus via contagion. During that first stage of the pandemic, before tests or vaccines for the virus became available, prevention was primarily a matter of avoiding actual physical contact and especially avoiding contact among larger groups of people, which could easily turn into what came to be known as “spreader events” where a single contagious person could spread the virus to many others who had not been physically present but had subsequent contact with those who did attend. Hygiene quickly became a controversial early preventive measure: while washing hands properly and frequently is still considered important to prevention, disinfecting surfaces (especially frequently touched surfaces in public areas) was eventually shown to be less effective in preventing covid-19 than avoiding exposure to viral particles suspended in the air (Pozzer et al., 2020; Zhang et al., 2022).

During 2020, implementation of mask regulations became one of the most controversial topics of the pandemic. Initially adopted in regions where air pollution levels and the 2009 SARS outbreak had already made mask wearing habitual, mask mandates were eventually introduced as public health policy at different points in time in various countries. The mandates varied widely over time and from country to country, partly due to covid-19 outcomes and partly for political reasons. An interesting point related to my argument in this article is that WHO did not initially recommend masking as a prevention measure. While the contentious issue of which type of mask to wear (cloth, surgical, FFP2, or its equivalent NK95) continued to be hotly debated throughout the pandemic, until as late as June 2020 WHO’s official position was that the potential risk reduction from wearing a mask was not high enough to justify the (assumed) corresponding increase in risky behaviors that would result from a sense of increased safety (ostensibly) afforded by mask wearing, as well as involuntary mistakes wearing the mask itself. It was only as the global death toll rose and the socioeconomic consequences of the pandemic became more pronounced that WHO finally issued masking recommendations.

Eventually, as testing became increasingly routine during the first year of the pandemic, test centers opened and offered free covid-19 testing, while rapid test kits were sold in pharmacies and online. Revisiting this earlier period of the pandemic brings the historically situated, circumstantial contingency of prevention measures to the fore: from 2020 to 2021, “prevention” primarily meant diminishing risk of exposure to virus particles by avoiding both physical contact and breathing in contaminated air. Vaccine safety and mask efficacy became strongly debated topics, in what ranged from politicized ethical debates to political controversies thinly disguised as ethical discussions. Safety and freedom, or security and risk, were now the foremost concepts mobilized in debates on how to endure a pandemic that quickly became a global public health emergency. Mental health issues resulting from loneliness and social isolation soon became an additional public health emergency, appearing in prominent debates across mainstream media along with discussions of the economic and social devastation of the pandemic (Brooks et al., 2020; Aknin et al., 2022; Luthra et al., 2023).

These perceptions constitute only one of many possible impressions of that first covid-19 year, before vaccines became widely available in wealthier nations in what is considered the Global North, and are based on my own situated reading across mainstream specialized scientific articles and reviews (especially in the fields of immunology, epidemiology, and public health), clinical studies, media reports, popular science, and what I have begun to call “critical covid-19 studies” to categorize a broad range of critical responses to the pandemic throughout the arts, humanities, and social sciences. Situated at the intersection of science and technology studies and cultural history, my analysis deploys a transdisciplinary methodology informed by poststructuralism, discourse analysis, and comparative literary analysis to bring together a seemingly disjointed assemblage of texts that are nonetheless connected by a sustained effort to document and study the covid-19 pandemic from multiple disciplinary locations. Using coverage of the scientific and social aspects of the pandemic in five key periodicals aimed at a general audience (*Nature, The New Yorker, The New York Times, The Guardian*, and *The Economist*) as a starting point, I proceeded to examine the vast scope of emerging clinical and biomedical literature on covid-19 as it appeared in highly specialized scientific journals alongside empirical, conceptual, and critical responses to the pandemic advanced in social sciences and humanities publications.

In this article, I consider how the pandemic affected overdeveloped affluent nations in North America and Western Europe in comparative transnational contexts, with a focus on the United States (where I lived from 2005 to 2013) and Germany (where I have lived since 2013). My analysis is unavoidably inflected by my own experiences as a permanent resident of Germany, an EU citizen, and a socioeconomically privileged, highly educated, employed, middle-class, (often read as) white person. Here I neither intend to offer a comprehensive study of covid-19 prevention measures nor present my argument as a final conclusion. Instead, I seek to show how these (now familiar) prevention measures encapsulate a particular approach to public health through which desirable future outcomes are achieved by what I call the logic of prevention: a specific thinking style embedded in a modern paradigm centered around large-scale surveillance and biosecurity rather than mutual aid, collective care, or community support. Creating, collecting, disseminating, preserving and studying covid-19 accounts that diverge from this one (substantially or subtly) is an important task in historicizing a pandemic that many wish was over and yet is still affecting the lives of so many on a global scale. At the time of this writing, the covid-19 pandemic may already seem to some like a distant historical event even while it remains central in the lives of people with disabilities, chronic illnesses, mental health conditions, or long covid, as well as my own current research and everyday life. In this article, I historicize the covid-19 pandemic by offering what I hope will be a nuanced critical overview of some key prevention strategies (namely distancing, masking, testing, and inoculating) adopted from March 2020 onwards, until pandemic restrictions were lifted in most countries by October 2022.

Obviously, pandemic measures provide an excellent case study for how governmental and scientific institutions routinely fail to provide adequate care and support on a local and global scale, and even more so when attempting to mitigate the socioeconomic consequences of a global pandemic. But my central argument is not simply that covid-19 prevention strategies exemplify a larger corpus of biosecurity and biosurveillance that is less invested in fostering or protecting the wellbeing of communities or individuals than it is in predicting, managing and controlling the health outcomes (understood in a strict biomedical sense) of citizens and populations. What interests me here is how, by enacting a particular logic, or to employ Ludwik Fleck’s phrase, a particular “thinking style” (1979, published 1935), covid-19 prevention measures demonstrate how the paradoxical ambivalence at the core of what I am calling the “logic of prevention” operates in concrete, specific settings – how it cures and harms, how it protects and damages, and how its anticipatory orientation inserts the future into the ongoing, ordinary present. This, at least, is how I hope this article can contribute to historicizing covid-19 prevention measures in comparative, transnational North American and Western European contexts. Ultimately, the history of artificial immunity is deeply entangled with the history of a now pervasive thinking style that became increasingly dominant across biomedical, military, and governmental interventions over the course of the twentieth century: the logic of prevention.

## The logic of prevention: anticipation, prediction, control

In *Dear science and other stories*, Katherine McKittrick (2021, p.12) uses the phrase “without knowing totally” to indicate the ways in which knowing can also demand an acknowledgement of what cannot be known (at least not yet), and harnessing what is yet to be known as a site of possibility (rather than as simply limiting). McKittrick is writing specifically about epistemologies and methodologies of Black studies, and how knowledge production must always grapple with its own incompletion, its own partiality, its own erasures, and in fact even cultivate them in a deliberate manner to enunciate the foundational presence of uncertainty, ambiguity, and opacity. The task of scientific investigation is then to (re)encounter facts, events, or data with curiosity and imagination, rather than exclusively in the hope of “knowing totally” – an impossible task that only demands the dulling of the senses, a reduction of what it means “to know” to the most empirical of data, “purified” from error and insulated from contextual location (Fiedel, Malich, Varino, 2019).

In this article, I mobilize the concept of the *pharmakon*, a term that has been perhaps somewhat overused in critical and cultural theory, as a container for the paradoxical relation of harm and cure to articulate their presence across measures to prevent covid-19. As historian of medicine Charles Rosenberg (2014, p.xii) points out in the “Foreword” to *Intolerant bodies: a short history of autoimmunity* by Warwick Anderson and Ian R. Mackay (2014), “the immune system has proved a seductive source of language and metaphor for a variety of philosophers and social theorists hoping to think with the body and about the body in society, to explore in contemporary terms ancient and enduring problems of the self and its relations.” It is these “enduring problems” that I am interested in examining in the context of the covid-19 pandemic. The preventive object-practices I consider, such as face masks, social distancing, testing, and vaccines, are all embedded within a thinking style I call “logic of prevention,” as they enact biomedical protocols of surveillance, security, and control explicitly developed to engender an anticipated future outcome, whether bound to practices of containment, harm reduction and/or risk management.

In this section, I want to consider how immunity to covid-19 works politically as well as clinically and scientifically. The paradox at the core of immunity is that in order to be immune, exposure to an agent that stimulates an immune response must first occur. That agent may be beneficial, innocuous, or harmful, or even deadly (in which case immunity will fail to be acquired) to the specific organism that encounters it. But the ways in which a specific organism responds to the presence of an agent, mounting a more or less severe immune response, will to a large extent determine whether the effect of the response becomes harmful, damaging the cells and tissues of vital organs, or constitutes a successful inoculation, protecting the organism “in the future.” Ultimately, the effects of these encounters are not in fact pre-determined, thus escaping western fantasies of control and mastery. Artificial immunity does not rely simply on vaccines as inoculation technologies, nor is acquired immunity exclusively dependent on exposure to a pathogen. Rather, immune responses are mounted by the physiological, cellular, and molecular processes of a specific body, over a specific period of time, continuously in contact with and affected by its material and social surroundings.

I engage with the realm of the viral itself as a *pharmakon* of sorts, whereby viruses have functioned as essential participants in the development of conditions amenable for multicellular non/human life to flourish. Normative models of health are necessarily informed by culturally and historically bound ideals of order and purity. How might a critical history shift normative assumptions about biomedicine, about health and immunity, and about individuality? That is, how does (historicizing) covid-19 prevention measures contribute to an expanded understanding of immunity as an individuated and collective process, both internally and externally mediated, at once physiologically, socially, materially, and ecologically bound?

Although in my book project on the covid-19 pandemic I frame my historical analysis from 1918 to 2022 by writing genealogically, I do not have a firm commitment to finding instances of historical repetition and similarity. Instead, I remain invested in how difference and divergence are also genealogically diffracted across events, even as I take a primarily historical approach informed by the history of science and medicine. Does the coronavirus pandemic begin with the first reported cases of severe respiratory clinical scenarios at the end of 2019, months before the disease was identified, recognized, and named by WHO? Does it begin with a (possible) nonhuman-to-human transmission of the virus or with the (purported) laboratory leak that infected the first human body, whether this led to the development of symptoms or not? Does it begin with the long history of coronaviruses inhabiting non/human life forms? Is there more to the covid-19 origin story, and should we probe deeper into how a virus carried by a number of mammals might have made its way into human bodies? Or should we pay more attention to the epistemological temporalities of viral models of pathogenesis within the history of immunological and biomedical knowledge production? Perhaps the multiple temporalities of the pandemic are better described in physiological terms, as viral particles enter a vast range of living human bodies through the mouth and nose, into bodies which are more or less hospitable, more or less likely to mount an immunological response, more or less capable of hosting it, more or less prone to forming alliances with it, more or less likely to sustain a full-blown immunological response of high fever and increased heart rate, potentially accompanied by a range of respiratory, cardiac, gastroenterological, neurological, and even dermatological symptoms.

As a plethora of authors and studies have shown, the covid-19 pandemic does not encompass only its devastating clinical consequences (Varino, 2021; Marzana et al., 2022; Milman, Lee, Neimeyer, 2022). Much like the aids crisis, its far-reaching effects also produced a pandemic of uncertainty: about the efficacy of masks, vaccines, and lockdown measures, about the accuracy of covid-19 tests, about the socioeconomic consequences of prevention measures, about the intricacies of covid-19 pathogenesis, about risk behaviors and the psychological consequences of isolation and social distancing. One of my central arguments is that we still must grapple with how prevention measures also have the capacity to harm, especially when they are designed and implemented within the scope of a biosecurity paradigm aimed at protecting “human” lives, rather than seeking to foster infrastructures so that interspecies ecosystems can thrive. Since becoming immune to a contagious disease like covid-19 requires interaction with either a virus or a vaccine, the predictive logic at the core of immunology is predicated on anticipating viral transmission patterns and/or vaccination rates. The concept of “herd immunity,” for example, permeated some covid-19 narratives as a positive future outcome of the pandemic to aspire to and work towards. But what would happen if prevention was dislocated from the immunological to the environmental realm, and thus from an anthropocentric to a non/human interspecies paradigm of interconnected life forms?

This shift in the logic of prevention is currently underway, with WHO’s official adoption of the One Health model of disease prevention in 2017 and various governmental initiatives worldwide which attempt to account for the influence of climate change and environmental factors on global public health (Hitziger et al., 2018; Zinsstag et al., 2021). The One Health model can be seen to function as an explicit attempt to prevent, contain, and manage future public health crises, particularly those generated by or coeval with climate change. By focusing on climatic and environmental phenomena, the model in fact offers a conceptual intervention that dislocates public health from modern science’s biomedical and clinical contexts to place them firmly within the scope of ecological concerns. In the case of the covid-19 pandemic (and future epidemics and pandemics), this translates into highlighting how habitat loss, deforestation, erratic weather patterns, pollution, dysregulated consumption, and frequent interspecies contact can affect human and nonhuman health, particularly through zoonotic disease transmission (Aarestrup, Bonten, Koopmans, 2021; Keusch et al., 2022; Holmes, 2022; Mallapaty, 2022).

In “Anticipation: technoscience, life, affect, temporality,” Adams, Murphy, and Clarke (2009) trace the anticipatory logic of predictive models at the core of algorithmic and statistical calculations, demonstrating how a constant state of anticipation focused on future outcomes and risk prediction comes to determine action in the present. In this manner, the development, recommendation, and implementation of prevention measures are often predicated on measuring (past) statistical frequency to predict potential (future) risk and foster safety. Within the scope of my book project, I show how covid-19 prevention measures participate in and produce what Adams, Murphy, and Clarke call a politics of temporality. In the following sections I consider how strategies of containment such as distancing and masking functioned in relation to propagation strategies, whereby exposure to viruses and vaccines eventually produced higher levels of immunity to covid-19.

## Containment versus propagation

In this section I turn to a comparative analysis of the physically oriented strategies of social distancing, quarantining, and masking as instances of what I am calling strategies of “containment,” with what I call physiologically oriented strategies of “propagation,” centered on deliberate exposure of large segments of the population to covid-19 vaccines and viral materials. The success of anti-vaccination rhetoric and movements cost millions of lives, but as many have claimed, there was also a widespread failure to publicly acknowledge and address very real public concerns about vaccine safety (Sowemimo, 2023; Schalk, 2022; Washington, 2020). Instead of summarily dismissing vaccine skepticism as a sign of ignorance or “selfishness,” I want to probe into the root causes of vaccine skepticism in overdeveloped wealthy nations like the United States and Germany, where covid-19 vaccines became widely available to most (though by no means all) segments of the population by the summer of 2021. Suspicion of the State, the law, the government, and the medical establishment became entangled with centuries-long hesitancy regarding inoculation. Resistance to modern medicine also played a role, with many vaccine skeptics favoring natural remedies and/or the body’s “natural” immune response.

Once it became clear that reaching herd immunity would be impossible as the highly contagious Delta variant caused more unnecessary and preventable deaths, there was an accompanying failure in scientific communication and public health outreach. The bulk of the blame for this failure cannot be easily attributed to scientists or health professionals, who may have used every outlet at their disposal to clearly articulate how and why covid-19 vaccines were indeed not only safe but also indispensable for saving lives. Although improvements in scientific communication and public outreach are undoubtedly necessary, I think that mainstream media and public policy share most of the responsibility in this case. For example, the long history of technoscientific advances that enabled the apparently “quick” development of covid-19 vaccines would not have been so surprising to large sections of the population had primary and secondary science education encompassed a more rigorous curriculum in the history of science and scientific education. Similarly, the ancient figure of the “village doctor” (or its contemporary equivalent) as a trusted authority could have done a lot to assuage and reassure people that vaccination was safe and urgently necessary. In many cases, such figures did serve in this role, from healthcare professionals to teachers and public figures who used their authority or expertise as a trustworthy source of information, from Anthony Fauci’s public outreach in the US to large-scale public health campaigns in Europe. But the increasing privatization of the health sector and consequent decay of public health systems across affluent overdeveloped nations such as the UK and the Netherlands, combined with the absence of a public health system in countries like the US, contributed significantly to delays in communication that could potentially have saved many lives as a prevention strategy.

In a sense, using communication as a prevention strategy is akin to the strategy of propagation encapsulated by vaccines. Instead of “containment,” they foster “propagation” of information, data, and ideas, alongside inoculation via vaccination or viral infection. Due to their non-pathogenic viral structure, the active components of covid-19 vaccines are not transmissible between bodies and thus cannot cause contagion. Their method of propagation is slow and highly specialized, and require inoculation via intramuscular injection administered by a trained healthcare worker (medical doctors, nurses, or other qualified health professionals), dedicated and/or repurposed sites (Tegel Airport in Berlin was utilized for vaccination, for example, while in other cities schools, city markets, gyms, and even pharmacies were converted into vaccination centers), and intricate infrastructure to manage scheduling, post-vaccination records, and documentation of side effects or symptoms. These propagation strategies relied on a sort of fluid, extended immunity that is pliable and responds to external conditions, communicable and networked across bodies, sites, data, and object-practices. Meanwhile, the propagation of insufficient, inadequate, or perhaps excessive amounts of information circulating in media outlets may have done a great deal of harm and impeded these propagation efforts to various degrees. In the following sections I consider how these two disparate orientations (containment and propagation) functioned as covid-19 prevention strategies within the context of pandemic management and risk reduction.

## Containing: distancing, masking, testing

Masking and distancing measures such as quarantine and isolation have long genealogies in the history of infectious disease. Long before the germ theory of disease became the dominant model across the life and health sciences, physical contact was already considered the main culprit in mechanisms of contagion, from leprosy and tuberculosis to diphtheria and poliomyelitis. Before pre-exposure prophylaxis (PReP) became widely available in affluent nations, aids prevention was limited to either using condoms as physical barriers or avoiding/minimizing sexual contact, particularly with people who might be asymptomatic but still contagious. As so many have pointed out, from immunology and public health expert Anthony Fauci (2020, 2021) to historian and public intellectual Achille Mbembe (2020), and linguist and queer theorist Mel Chen (2021), while in many ways the covid-19 pandemic was unprecedented, a great deal about it is uncannily familiar, with deep historical lineages into the distant and not-so-distant past. It does not take much probing to find various historical precedents. As Chen (2012, 2021) has observed, face masking practices may recall swine flu, high pollution levels, chemical warfare, or even science fiction scenarios of climate calamity. Masking is by no means a neutral, straightforward practice: covering the face invokes and reenacts a vast range of culturally and historically specific symbolic meanings, including biomedical imaginaries, outbreak narratives, moral panics, and a cataclysmic state of emergency. I use the term “covid-19 masks” to indicate the broad range of masks worn by the general population (with some models also used by healthcare professionals, such as surgical masks), from the makeshift swaths of cotton cloth with a stitched-on elastic ribbon which were common at the beginning of the pandemic to the FFP2 masks that became the legally required standard model in Germany and KN95 filtering masks and surgical masks.

The intense controversies that emerged from masking mandates were often formulated based on the commonly evoked rhetoric of “freedom,” “choice,” and “nature,” as though there were shared agreement on what constitutes proper “human” embodiment. Instead of presuming that certain forms of embodiment are “better” (i.e. more “natural” and, therefore, benign) than others, it might have been more useful for those mobilizing against mask mandates to clarify which forms of embodiment they preferred and why. For example, claiming a preference for unmasked faces and unvaccinated bodies might be a way to also state whose lives are deemed valuable and whose deaths are considered necessary (or at least justifiable) based on racist, ableist, ageist, and classist regimes. On the other hand, it might express a dislike for state-sanctioned measures based on scientific evidence, and perhaps even encapsulate a desire to actualize the evolutionary ideal of human bodies properly adapted and responsive to their surroundings, capable of generating appropriate immune responses that do not jeopardize the survival of the individual.

The logic of prevention unavoidably assigns economic value to certain courses of action as potentially beneficial for the nation-state, based on their future implications. Whether a course of action is preferable or recommended is therefore based on eventual gains, and the costs calculated in relation to those gains. The costs of masking (inconvenience, discomfort, time, money, waste), according to those who oppose it, may not be offset by the value of saving lives and avoiding disease. In contrast, governments and public health organizations determined that the socioeconomic value of keeping hospitalizations low, decreasing mortality rates, and lowering infection numbers were priorities worth the overall costs. The logic of prevention, as demonstrated by pandemic measures, cannot help but replicate and indeed reenact biosecurity concerns, which assign economic value to protecting and cultivating the health and wellbeing of certain segments of the population while leaving others to perish instead of centering on human rights and social justice values like care, accountability, and mutual responsibility.

## “Recovered, tested, vaccinated:” propagation as preventive transmission

In this section, I consider the “3G Regulations” adopted in Germany as exemplifying an orientation towards propagation in the prevention of covid-19. The term 3G derives from the German words for recovered (*genesen*), vaccinated (*geimpft*), and tested (*getestet*), all starting with “ge” as a past participle of the respective verbs transformed into adjectives, and functioned as both acronym and mnemonic in public health campaigns. Drawing from the occurrence of a past event contiguous with the present, the choice of verb tense encapsulates the hope that the present and immediate future are reasonably likely to be characterized in terms of past occurrences. As such, based on information concerning the recent past, the body is presumed not to be contagious “for the time being,” lacking enough viral load to be able to transmit covid-19 to another living body because it has been inoculated, exposed to the virus, or recently tested negative (during the 3G regulation implementation period in 2021-22, only PCR and rapid antigen tests administered at official covid-19 test centers were considered valid, as opposed to rapid antigen testing at home).

Historically, the germ theory of disease has been politically mobilized to place the blame for illness and disease on “foreign others.” Similarly, the notion of disease as “preventable” through hygiene, fitness, or nutritional regimes is predicated on eradicating supposedly “pathogenic others,” from toxins and germs to processed sugars and saturated fats. Ed Cohen’s (2009) notion of a “body worth defending” is concomitant with the notion of a nation-state worth preserving, but incommensurate with social justice values that render all (human) lives valuable, since for the nation-state the living bodies that count are always already normatively prefigured as healthy, able, and autonomous. This sanctification of the body requires that impurity be attributed to others – pathogenic others, whose normative autonomy has been compromised by infectious or chronic disease, by disability or neurodivergence, by ways of being and doing that are not aligned with the project of economic and political reproduction. The bodies worth defending, according to this neoliberal paradigm, are those that can withstand invasion and attack by a foreign “other” arriving from a hazardous “outside” and still respond properly by mounting an adequate immune response, thus becoming something along the lines of the figure of the “salted horse:” a living body that has successfully overcome infectious disease and grown stronger as a result. Long covid, for example, supposedly presents an “anomaly” in spite of its high frequency, with millions of people still affected at the time of this writing. Its symptomatology is so diverse it poses a medical puzzle, with a range of symptoms spanning respiratory, cardiac, gastric, neurological, dermatological, and psychological conditions. Baffling for scientific studies, it seems that only polyvalent broad categories like “sequelae” and “convalescence” can adequately account for the multiple variations of long covid across clinical scenarios.

The *pharmakon* is thus a useful concept for understanding how vaccines work not only in the sense that a remedy may be poisonous, but also in the sense that the therapeutic function of the cure is inextricably dependent on its capacity to harm. In fact, without some degree of harmful impact, no therapeutic outcome is possible via inoculation. The logic of inoculation remains tricky, whereas the logic of treatment via medication appears much more straightforward despite the risk of side effects. Antibiotics, for example, are supposed to annihilate the “enemy” and their biocide function is easier to grasp, damaging targeted pathogenic bacterial life forms while also harming important beneficial bacteria. Episodes of antibiotic resistance are becoming more frequent, an issue considered to be a major global public health concern by WHO and other public health organizations. Conversely, the harm caused by antibiotics has historically received much less attention across clinical studies, popular science, and mainstream media, with biomedical research primarily focusing on reported side effects or allergic reactions. The potential side effects of covid-19 vaccines, however, cannot be simply relegated to the category of rare unrelated secondary effects but instead have to a certain extent jeopardized the efficacy of generously funded vaccination programs in countries like Germany and the US, where vaccine skepticism and anti-vaccination movements remain strong. Public health education efforts have for the most part failed to articulate how a cure can be paradoxically harmful, in individual cases as well as across large-scale public health programs, and how vaccines must cause some degree of harm to a living body in order to elicit an immune response.

Even though inoculation against covid-19 was conceived from the start as a global public health project, economic and sociocultural specificity made it extremely difficult to reach swaths of the population that were already suspicious of state measures, having been historically marginalized and unable to access healthcare, particularly in the US. Unquestionably, vaccination programs are embedded within vast economic networks that are imbricated with all the flaws and limitations of knowledge production influenced by economic, technological, linguistic, social, and material factors. As such, both biomedical research and clinical practice must make greater efforts to avoid reproducing the social inequalities that have historically shaped them and whose exclusionary structures they replicate. But much like viral variants, biomedical and clinical practice are also capable of variability – to what extent and how radically remains to be seen. Minor and major changes in biomedical research, public health programs, and clinical practice can massively impact large segments of the population and fundamentally change how marginalized groups and communities cope with large-scale calamities like climate change, oil spillages, or a global pandemic.

## Final considerations: left to die

As Eula Biss (2014, p.5) points out, “immunity is a myth.” As a myth, it propels not only much of biomedical research but many of the daily decisions, big and small, private and public, individual and collective, made by the citizens of affluent, overdeveloped nations. As a myth, it is an ancient one. As a twenty-first century utopia of sovereign power over our embodied lives, immunity has come to do a tremendous amount of political and conceptual work. In my current book project, I propose a genealogy of the covid-19 pandemic by focusing the development of covid-19 vaccines in the context of twentieth-century histories of inoculation. The concept of the *pharmakon* has allowed me to formulate the myriad safety and security issues galvanized by covid-19 vaccination narratives, models, metaphors and object-practices. Covid-19 vaccines encapsulate various characteristics of the *pharmakon*, pointing to the ambivalent function of any remedy as containing both harmful and beneficial effects. This ambiguity has been successfully deployed by movements that oppose covid-19 vaccination and is also evident in the rhetorical strategies mobilized in the vaccine skepticism which is still widespread. As biotechnological interventions that demonstrate the plasticity of bodies and immune systems, vaccines as artificial immunity are emblematic of the uncertainty at the core of biomedical knowledge production. I seek to engage with the realm of the viral itself as a *pharmakon*, whereby virions have functioned as essential participants in the development of conditions amenable for multicellular (non/human) life to flourish and their pharmaceutical and genetic applications have been widely studied.

In this article, I have examined how covid-19 prevention measures (distancing, masking, testing, and inoculating) have shaped a *pharmakon* of artificial immunity that encompasses the paradoxical ambivalence of cure and harm. How might we build a more therapeutic environment, socially, materially, politically, ethically, epistemologically? The covid-19 pandemic has relied on a range of preventive strategies that, I argue, might reduce the pandemic to an exclusively biomedical event, and equate public health with biosecurity. In critically engaging with ideas about planetary health in the Anthropocene age, along the lines of WHO’s One Health model, it becomes clear that pandemic prevention measures demand a model of immunity that accounts for its variability and denaturalize its mechanisms, showing how immunity processes are embedded and participate in political, material and sociocultural structures. Citizens of affluent nations in the Global North have grown used to internally oriented solutions for health optimization predicated on a biomedical model of individualized health. During the first decade of the aids crisis, Act Up’s slogan “Drugs into bodies” encapsulated a biomedical logic that is no longer applicable in any straightforward manner, given the vast range of deadly environmental conditions that condemn some living bodies to death while protecting the vitality of others.

It is crucial to come to terms with the decidedly colonialist, white supremacist, patriarchal, ableist legacy of scientific enterprise. The logic of eugenics still pervades much biomedical knowledge production. In heavily industrialized, technocratic, late capitalist societies, those of us with advanced degrees and higher levels of socioeconomic privilege do have a responsibility to drastically decrease the miles we fly, the meat products we eat, the water we waste, and the goods we consume. But much more importantly, we must find ways to collectively organize to demand implementation of urgent regulations on industrial, military, and nuclear waste. The way that viruses circulate and enter our living bodies is obviously not intended to teach us anything, but can serve to demonstrate yet again how extreme weather, deforestation, habitat loss, rapid species extinction, and nonhuman animal exploitation have very real consequences for the health and survival of humans in the Anthropocene. Learning this lesson is not hard, but acting upon it certainly is. And ultimately, our collective ability to act will determine how earthly life might persevere or perish.
